# The Role of Imaging Techniques to Define a Peri-Prosthetic Hip and Knee Joint Infection: Multidisciplinary Consensus Statements

**DOI:** 10.3390/jcm9082548

**Published:** 2020-08-06

**Authors:** Carlo Luca Romanò, Nicola Petrosillo, Giuseppe Argento, Luca Maria Sconfienza, Giorgio Treglia, Abass Alavi, Andor W.J.M. Glaudemans, Olivier Gheysens, Alex Maes, Chiara Lauri, Christopher J. Palestro, Alberto Signore

**Affiliations:** 1 Gruppo di Studio SIOT Infezioni-Clinica San Gaudenzio-Novara-Gruppo Policlinico di Monza, University of Milan, 20100 Milan, Italy; carlo.romano@unimi.it; 2 Clinical and Research Department for Infectious Diseases, National Institute for Infective Diseases “L. Spallanzani”, 00144 Rome, Italy; nicola.petrosillo@inmi.it; 3 Radiology Unit, AOU Sant’Andrea, 00189 Rome, Italy; giuseppe.argento@uniroma1.it; 4 IRCCS Istituto Ortopedico Galeazzi, 20161 Milan, Italy; io@lucasconfienza.it; 5 Department of Biomedical Sciences for Health, University of Milan, 20123 Milan, Italy; 6 Nuclear Medicine and PET/CT Center, Imaging Institute of Southern Switzerland, Ente Ospedaliero Cantonale Via Lugano 4F, CH-6500 Bellinzona, Switzerland; giorgiomednuc@libero.it; 7 Department of Nuclear Medicine and Molecular Imaging, Lausanne University Hospital and University of Lausanne, 1011 Lausanne, Switzerland; 8 Division of Nuclear Medicine, Department of Radiology, Hospital of the University of Pennsylvania, Philadelphia, PA 1904, USA; abass.alavi@pennmedicine.upenn.edu; 9 Department of Nuclear Medicine and Molecular Imaging, University of Groningen, University Medical Center Groningen, 9713 GZ Groningen, The Netherlands; a.w.j.m.glaudemans@umcg.nl; 10 Department of Nuclear Medicine, Cliniques Universitaires Saint-Luc, 1200 Brussels, Belgium; olivier.gheysens@uclouvain.be; 11 Department of Nuclear Medicine, AZ Groeninge, Kortrijk Belgium and Department of Imaging and Pathology @ KULAK, KU Leuven campus Kulak, 8500 Kortrijk, Belgium; alex.maes@azgroeninge.be; 12 Nuclear Medicine Unit Department of Medical-Surgical Sciences and of Translational Medicine, Faculty of Medicine and Psychology, “Sapienza” University of Rome, 00161 Rome, Italy; chialau84@hotmail.it; 13 Department of Radiology Donald and Barbara Zucker School of Medicine at Hofstra/Northwell, Hempstead, NY 11549, USA; palestro@northwell.edu

**Keywords:** prosthetic joint infection, nuclear imaging, SPECT/CT, PET/CT, radiology

## Abstract

Diagnosing a peri-prosthetic joint infection (PJI) remains challenging despite the availability of a variety of clinical signs, serum and synovial markers, imaging techniques, microbiological and histological findings. Moreover, the one and only true definition of PJI does not exist, which is reflected by the existence of at least six different definitions by independent societies. These definitions are composed of major and minor criteria for defining a PJI, but most of them do not include imaging techniques. This paper highlights the pros and cons of available imaging techniques—*X*-ray, ultrasound, computed tomography (CT), Magnetic Resonance Imaging (MRI), bone scintigraphy, white blood cell scintigraphy (WBC), anti-granulocyte scintigraphy, and fluorodeoxyglucose positron emission tomography/computed tomography (FDG-PET/CT), discusses the added value of hybrid camera systems—single photon emission tomography/computed tomography (SPECT/CT), PET/CT and PET/MRI and reports consensus answers on important clinical questions that were discussed during the Third European Congress on Inflammation/Infection Imaging in Rome, December 2019.

## 1. Introduction

The definition and the diagnosis of peri-prosthetic joint infection (PJI) and, more generally, of implant-related infections, remains a challenge of modern orthopaedics.

In fact, while it seems relatively straightforward to diagnose an infection in the presence of a draining sinus, an exposed implant, or classical signs and symptoms of an acute inflammatory process, the differential diagnosis between septic and aseptic implant failure becomes much more challenging when unspecific clinical symptoms—most often a variable degree of pain and reduced function—are reported, and laboratory tests yield nonspecific or conflicting results.

Clinical presentations of peri-prosthetic infection are extremely varied, ranging from the acute, high-grade inflammatory cases to the subclinical low-grade ones [1,2,3]. The lack of a single accepted reference test or benchmark makes the evaluation and comparison of the diagnostic accuracy of both old and new markers, as well as other diagnostic tools, particularly difficult [4,5,6].

The diagnostic challenge is mirrored by the absence of a universally accepted definition of PJI. In the last decade, at least six different definitions of PJI have been released by well-respected scientific societies, including the Musculo-Skeletal Infection Society (MSIS) [7], the Infectious Disease Society of America (IDSA) [8], two International Consensus Meetings [9,10,11], the European Bone and Joint Infection Society (EBJIS) [1], (Table 1) and, more recently, the World Association against Infection in Orthopaedics and Trauma (WAIOT) (Table 2) [12].

These six definitions differ greatly in their diagnostic criteria, scoring systems and reference values (Table 1 and Table 2), while even the most complex scores may result as “inconclusive” in a given patient [10]. Of note, with the exception of the WAIOT’s definition, all other proposed definitions of PJI include only a selection of diagnostic tests while systematically excluding any role of imaging, in spite of their reported diagnostic value (Table 2) [14]. In doing so, none of them provide a clear scientific explanation for this exclusion, while on the other hand, it is a common observation that most clinicians do prescribe some imaging investigations when dealing with a (suspected case of) PJI. In this complex panorama, to further understand the role of imaging techniques in the diagnostic protocol of peri-prosthetic joint infections, a multidisciplinary group met from December 9 to 12, 2019 in Rome, during the Third European Congress on Inflammation/Infection Imaging.

The results of the discussions, held during those days and thereafter through online consultations, are reported here in the form of clinical questions with consensus answers. These are also based on the previously published Italian Guidelines to Diagnose Peri-Prosthetic Joint Infections [15] and joint European guidelines on PJI published by European Association of Nuclear Medicine (EANM), European Bone and Joint Infection Society (EBJIS) and European Society of Radiology (ESR), with the endorsement of European Society of Clinical Microbiology and Infectious Disease (ESCMID) [14,16], to which we refer that contain details about all imaging modalities.

## 2. Assessment Parameters of Peri-Prosthetic Joint According to PJI Definitions

Current PJI definitions rely on four diagnostic classes of investigations: (1) clinical presentation, (2) serum and synovial markers, (3) imaging techniques and (4) microbiological and histological findings (Table 1 and Table 2).

Concerning clinical presentation, the presence of a draining sinus or of an exposed implant is considered as pathognomonic or highly specific by all the available definitions [7,8,9,10,11,12]. However, this sign may be totally absent in more than 70% of peri-prosthetic joint infections, thus featuring a quite low sensitivity [17].

Serum and synovial fluid markers are variably included in all the available PJI definitions, apart from the one released by IDSA, while the proposed EBJIS definition only considers synovial leukocyte cell count. No single biomarker has been shown to be 100% accurate in diagnosing PJI, and therefore all definitions introduced a scoring system based on combining the results of different tests. These scoring systems not only vary greatly among the definitions, but also differ in cut-off values that are chosen for the various definitions, which limits their comparability. Furthermore, most of the definition systems acknowledge the fact that serum and synovial biomarkers results should be interpreted with caution within the first three months after surgery and in patients under antibiotic treatment or patients with concomitant systemic inflammatory diseases.

Concerning imaging, no available PJI definition includes any of these investigations, except for the recently released WAIOT definition (Table 2).

The WAIOT definition, validated in a large clinical, multi-institutional and international trial [13], includes only two imaging techniques, 99metastable Technetium (^99m^Tc)-bone scan and ^99m^Tc-leukocyte scan, chosen according to the available literature, respectively as a ‘rule out’ and ‘rule in’ test to define PJI. In this regard, it should be noted that the WAIOT definition provides a set of rule out and rule in tests, among which the clinician is left free to choose. The final definition is based on the relative balance of positive rule in tests and negative rule out tests. Microbiological and histological findings are considered relevant investigations by all the available definitions to confirm PJI. More specifically, positive cultures, even if criteria and recommendations vary across definitions, are considered pathognomonic by all of the classification systems examined in Table 1 and Table 2. However, limitations do apply with regard to the interpretation of a single positive culture and for suboptimal procedural investigations. In fact, falsely negative cultures are reported in approximately 20% of PJIs, according to a recent review [18]. Therefore, microbiological sampling, transport and processing should be performed according to the best available microbiological standards, which includes the preparation of four to six peri-prosthetic tissue cultures and the analysis of the removed implant, transported in closed systems processing with antibiofilm techniques (sonication or dithiothreitol) and with prolonged cultures. In selected cases, genomic pathogen identification may also be advisable [19].

Similarly, histology is ranked among the most specific examinations to differentiate a PJI from other causes and is highly scored or plays a confirmatory role in five out of six of the examined PJI definitions, even if its sensitivity may by be as low as 57% and it may be prone to interpretation bias, according to the experience of the pathologist [20].

## 3. Conventional Techniques for Diagnosis of PJI

The first diagnostic imaging modality is generally conventional radiography.

*X*-ray examinations are the standard examination to perform after arthroplasty and for follow-up to assess the presence of displacement, mobilization of the implant components, periprosthetic bone resorption and other causes of pain.

However, diagnostic performance of conventional radiography in detecting PJI is very low. Furthermore, conventional radiography may show demineralization only when more than 30–50% of bone mass has been lost, and abnormalities of bone around the implant are usually non-specific for infection. In addition, up to 50% of conventional *X*-ray exams give negative results.

Regarding ultrasound (US), disputable results have been reported for the detection of PJI. US may be used to guide aspiration procedures of infectious materials in PJI and can be effectively used to evaluate peri-prosthetic fluid collections, attempting to differentiate abscesses from aseptic collections [21], and to track the presence of sinus tracts within soft tissues. The main advantages of US are its wide availability, low cost, the possibility to perform it bedside and repeated imaging without radiation burden [21].

**Figure 1:**
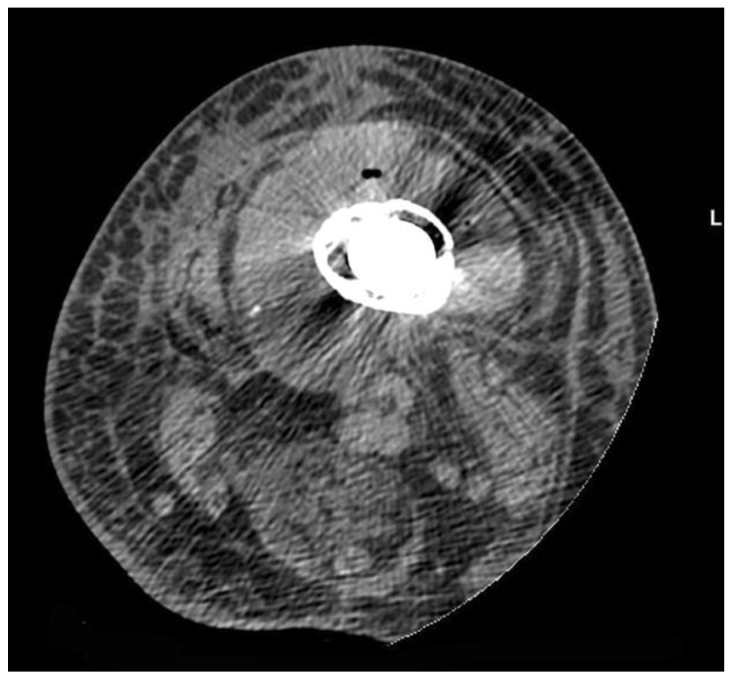
Computed tomography (CT) features: axial scan showing a fluid collection with increasd density surrounding infected bone with a prosthetic implant. Note the swelling and hyperdensity of soft tissues due to edema.

Computed tomography (CT) has been reported to have a good diagnostic performance in the detection of PJI, with accuracy of up to 84% (Figure 1). CT is also the imaging modality of choice perform image-guided bone biopsies [22].

Most papers dealing with magnetic resonance imaging (MRI) in the field of PJI have been focused on technical feasibility and metallic artefact reduction.

**Figure 2:**
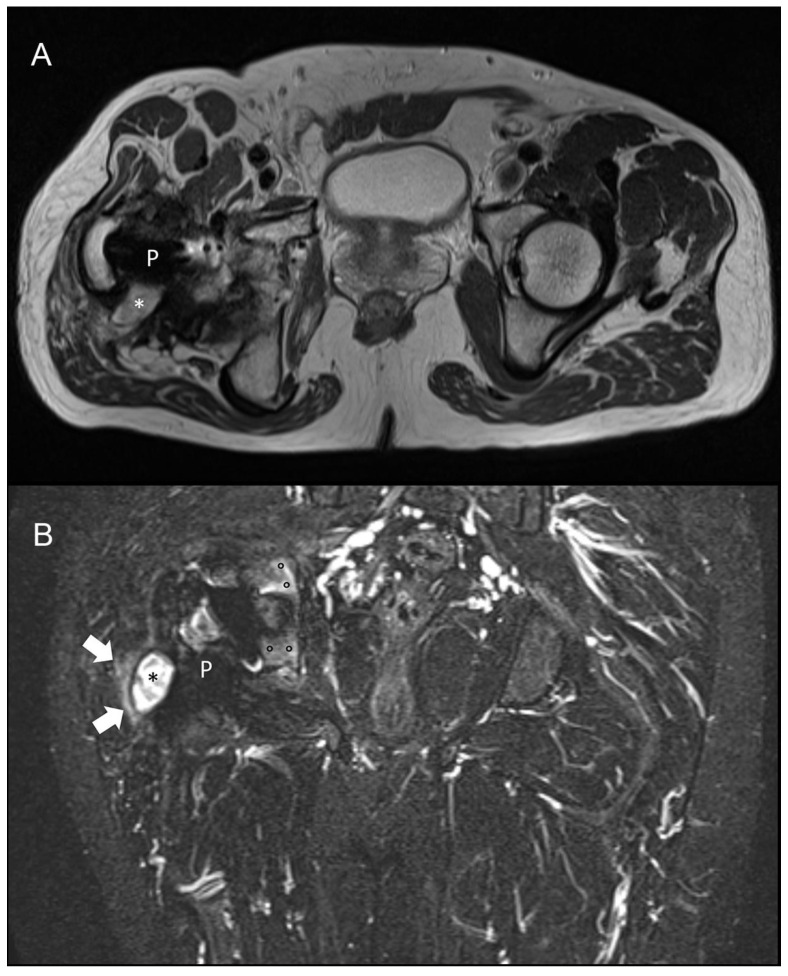
Magnetic resonance imaging (MRI) of a 58-year-old patient with culture-proven infected right hip prosthesis. (**A**) T2-weighted axial and (**B**) short-tau inversion recovery coronal scan show hyperintense synovitis (asterisks), extra-capsular edema (arrows), and bone edema (circles). Metallic artefact is limited to the implant only (P) and does not obscure the findings of infection.

For knee arthroplasty, MRI has been shown to be highly sensitive (92%) and specific (99%) for diagnosing PJI. Only one paper has been published in patients with hip arthroplasty showing that the presence of periosteal reaction, capsule edema, and intramuscular edema has a high accuracy for evaluating PJI [23], (Figure 2). Similar to US, MRI has the advantage of not using ionizing radiation or contrast agents [24,25,26].

Both CT and MRI may be useful to document the extent of bone lesions as well as abnormalities in the articular space and, therefore, they may help the surgeon in planning the most appropriate strategy. Moreover, US and CT are extremely useful for performing (when feasible) fluid aspirations, thus representing an important tool in the diagnostic work-up of PJI.

## 4. Nuclear Medicine Techniques for Diagnosis of PJI

Several imaging techniques can be used to evaluate PJI including bone scintigraphy, radio-labelled white blood cell (WBC) scintigraphy (with or without combined bone marrow scintigraphy), anti-granulocyte antibody scintigraphy, and fluorodeoxyglucose positron emission tomography ([^18^F]-FDG-PET).

Both planar and tomographic acquisitions, with single photon emission tomography (SPECT), can be performed and the use of hybrid modalities such as SPECT/CT or PET/CT increases the diagnostic accuracy in terms of the exact location and extent of the infectious process. Importantly, scintigraphic techniques by gamma camera are not affected by metallic hardware; PET/CT may present some artefacts.

### 4.1. Bone Scintigraphy

Bone scintigraphy is usually performed after the injection of ^99m^Tc-labelled diphosphonates and a three-phase bone scintigraphy can be performed to assess early perfusion, diffusion, and late bone uptake. The uptake of these tracers is usually related to bone remodelling. After a prosthetic implant, the periprosthetic bone is obviously damaged and a remodelling process will occur in the months following surgery.

This remodelling process is more evident for bio-inductive prostheses compared to cemented prostheses. The main advantage of bone scintigraphy is its very high sensitivity (when negative, it rules out an infection with high certainty), but this method is accompanied by a low specificity for PJI.

Conversely, this method may be able to detect bone abnormalities in case of prosthetic mobilization, particularly if a hybrid SPECT/CT technique is used. Recently, the EANM Bone and Joint Committee has published procedural guidelines on how to perform this modality best for each pathology [27].

### 4.2. White Blood Cell Scintigraphy

**Figure 3:**
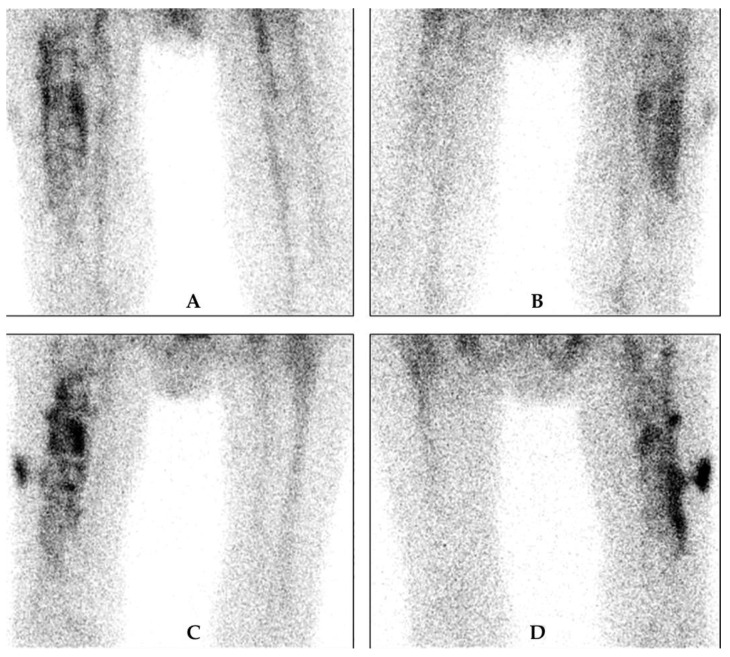
Example of ^99m^Tc- hexamethylene-propyleneamine oxime (HMPAO)-WBC scintigraphy in a patient with suspected PJI. Upper row: delayed images, (**A**) anterior and (**B**) posterior view, and lower row: late images, (**C**) anterior and (**D**) posterior view. There is increase in intensity and in size between the delayed and late images, which is very suspicious of a PJI. There is also extension of the infection to the peri-prosthetic soft tissue at the lateral side.

**Figure 4:**
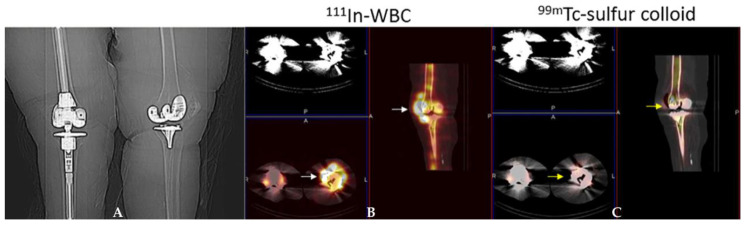
Periprosthetic infection left knee arthroplasty. Scout radiograph (**A**) demonstrates bilateral knee arthroplasties. The right is a revision arthroplasty and the left is a primary implant. On the ^111^In-WBC single photon emission computed tomography/computed tomography (SPECT)/CT) (**B**) there is abnormal labeled leukocyte activity along the anterior aspect of the left knee arthroplasty (white arrows). On the ^99m^Tc-sulfur colloid SPECT/CT (**C**), there is no corresponding activity in this region (yellow arrows) and therefore the study is positive for infection.

WBCs can be labelled with ^99m^Tc- hexamethylene-propyleneamine oxime (HMPAO) (Figure 3) or ^111^In-oxine (Figure 4). The labelling method, image acquisition and interpretation are regulated by several national rules and guidelines [28,29,30].

Taking into account the different biodistribution and kinetics of radio-labelled WBCs in blood, bone-marrow, infection and sterile inflammation, images should be acquired at three different time points with decay time-corrected acquisition: “early images” (within 30 min and 1 h after radiopharmaceutical injection), “delayed images” (between 2 h and 4 h after radiopharmaceutical injection) and “late images” (between 20 h and 24 h after radiopharmaceutical injection). Even though the diagnosis of a PJI is made on planar images (increase in uptake or size between the delayed images at 3–4 h and the late images at 20–24 h), tomographic images are recommended in case of positive planar images to assess the exact location and extent of the infectious process. Using these image acquisition parameters and interpretation criteria, this technique reaches a high sensitivity and specificity, as a recent multicenter study has shown [31]. The overall diagnostic accuracy of this technique exceeds 90% for PJI and this method constitutes the gold standard imaging technique for diagnosing PJI.

### 4.3. Anti-Granulocyte Antibody Scintigraphy

^99m^Tc-labelled monoclonal antibodies (mAbs) may be used as an alternative to WBC scintigraphy to evaluate PJI. Besilesomab is a full size anti-granulocyte mAb produced in murine cells and designed to attach to the non-specific cross-reacting antigen (NCA)-95 antigen localized on the surface of granulocytes. Sulesomab is an antigen-binding mAb fragment designed to target the NCA-90 antigen on the surface of granulocytes. For radio-labelled mAbs, imaging protocols differ between complete and fragmented antibodies [32]. The acquisition protocol for full length antibodies (Besilesomab) is similar to WBC scintigraphy. The best time point for SPECT images is at 16–24 h post injection, similarly to WBC, but an early scan can also be performed if required.

In contrast, with ^99m^Tc-sulesomab, planar images should be performed 1 h and 4–6 h post injection due to the faster clearance of the fragmented antibody [30].

### 4.4. Bone Marrow Scintigraphy

Bone marrow scintigraphy is usually recommended in addition to WBC scintigraphy in equivocal cases. The radiopharmaceutical used is ^99m^Tc-colloids (colloids greater than 500 nm) that enables visualization of the bone marrow (thus distinguishing expanded bone marrow from sites of leukocyte accumulation). About 185 MBq of ^99m^Tc-colloids are intravenously injected and planar scintigraphic images of the region of interest are usually acquired after a minimum of 20–30 min and a maximum of 6 h post injection [30]. Concordant findings between both techniques rule out an infectious process while discordant findings (uptake on WBC scintigraphy without corresponding uptake on bone marrow scintigraphy) are highly suggestive of an infection.

### 4.5. FDG PET/CT

**Figure 5:**
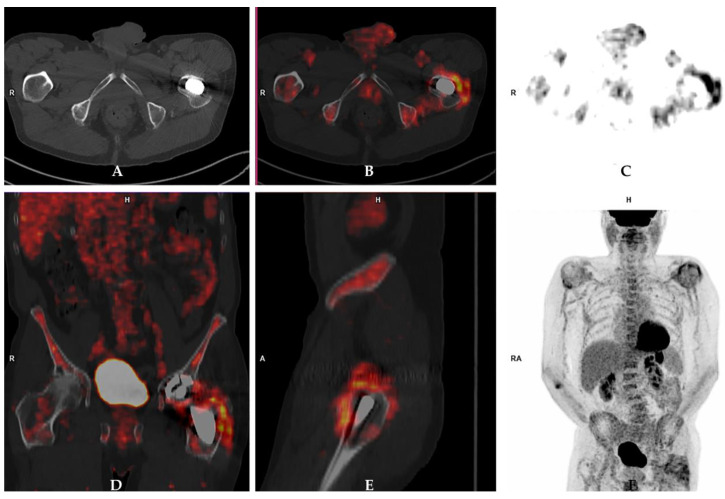
Prosthetic joint infection detected by 18Fluorine-fluorodeoxyglucose positron emission tomography/computed tomography ([^18^F]-FDG-PET/CT) in a 72-year-old male patient who underwent left hip arthroplasty four years before the PET/CT scan. [^18^F]-FDG-PET/CT images (**A**: axial view of CT scan; **B**: axail fused images; **C:** axial PET images; **D**: coronal [^18^F]-FDG-PET/CT view; **E**: sagittal [^18^F]-FDG-PET/CT view; **F**: maximum intensity projection [^18^F]-FDG-PET images) showed increased radiopharmaceutical uptake in the periprosthetic bone and soft tissues at the level of the femoral component of the prosthesis. These findings were indicative of prosthetic joint infection, which was confirmed by further examinations including microbiological culture.

Although [^18^F]-FDG-PET/CT offers several advantages over WBC scintigraphy (more convenient for the patient, no need for cell labeling, whole procedure takes less than 2 h), looking at the available published data so far, it is unclear whether [^18^F]-FDG-PET may offer significant advantages over radio-labelled WBC or anti-granulocyte monoclonal antibodies for the evaluation of PJI [33,34]. Different interpretation criteria for PJI have been proposed by Reinartz et al. [35], Chacko et al. [36], Love et al. [37], Familiari et al. [38] and Stumpe et al. [39], but all these studies led to an overall accuracy of <90% with conflicting results amongst studies [40,41]. In any case, visual interpretation is generally more reliable than quantitative (SUV) analysis, which is currently not recommended (Figure 5).

### 4.6. Hybrid Imaging Techniques

Hybrid imaging modalities combining functional and anatomical data have significantly increased the accuracy of conventional nuclear medicine modalities by reducing the number of doubtful cases. The hybrid imaging approach leads to a more accurate assessment of both localization (soft tissue vs. bone vs. both) and disease extent.

SPECT/CT is nowadays often performed as an integral part of a conventional WBC/mAb scintigraphy in order to better localize the uptake into bone or soft tissues and to accurately assess the extent of the infection [42,43,44,45,46]. [^18^F]-FDG-PET/CT can be considered as a first-line diagnostic tool for evaluating patients with inflammatory diseases and/or fever of unknown origin, according to evidence-based data [41]; in cases of spondylodiscitis and fungal infections, its role has also been well described.

More recently, the introduction of PET/MRI has emerged as a powerful diagnostic tool, but its value in PJI has not been systematically addressed. The general advantages of MRI compared to CT include a better evaluation of soft tissue and a lack of radiation burden. In addition, MRI sequences that avoid artefacts from metallic implants are now widely available [23,24,25,26,47,48,49,50,51].

Finally, it is worth mentioning that one should always keep in mind that the final decision for a particular imaging technique will be highly dependent on the local availability, time, cost and expertise.

## 5. Clinical Questions and Consensus Answers

At the Third European Congress on Inflammation/Infection Imaging, held in Rome on December 2019, there were several sessions dedicated to radiological and nuclear medicine imaging of prosthetic joint infections. The round table discussions, with clinicians and specialists in infective disease, were very much animated in particular with regard to the role of CT vs. MRI and FDG-PET/CT vs. radio-labelled WBC. Below are the main points raised by orthopedic surgeons and the answers provided by radiologists and nuclear medicine physicians.

### 5.1. What Is the Role of Conventional X-ray to Diagnose a PJI?

There is no role of plain films for differential diagnosis of PJI. Nevertheless, an *X*-ray image can be very useful to evaluate other concomitant problems, the degree of loosening, bone reabsorption, fractures, etc. that may all help for the interpretation of images obtained by other modalities, particularly non-radiological modalities [52]. For this reason, conventional radiography remains the first imaging modality in patients with suspected PJI and for their follow-up [14].

### 5.2. What Is the Role of Ultrasound to Diagnose a PJI?

Data on the use of US to diagnose PJI are scarce and conflicting [14]. At present, US is mostly used to guide joint aspiration or biopsy to perform microbial culture [21].

### 5.3. What Is the Role of CT to Diagnose a PJI?

Whenever CT is used to diagnose a PJI, artefacts caused by the interaction between *X*-ray beams and metallic hardware should be reduced by suitable software and techniques [16]. Joint capsule distension and the presence of fluid collections in the soft tissues surrounding a hip implant showed 100% sensitivity, 87% specificity, and 89% accuracy when at least one soft tissue abnormality was used as an infection criterion, and 83% sensitivity, 96% specificity, and 94% accuracy when joint distension was used as infection criterion [22]. CT may be used to more effectively diagnose bone resorption and bone lucency around the implant compared to plain films, however this may not be considered a reliable parameter to differentiate between infection and other reasons for implant failure [22]. CT is useful for guiding biopsies and fluid aspiration.

### 5.4. What Is the Role of MRI to Diagnose a PJI?

The advent of prostheses made with less ferromagnetic alloy materials and the introduction of metal artefact reduction sequences (MARS), slice encoding for metal artefact correction (SEMAC), and multi-acquisition with variable-resonance image combination (MAVRIC) has pave the way for the use of MRI in patients with joint prosthesis, limiting the artefacts to the area of the implant itself. However, published data about the role of MRI to diagnose PJI are still very limited. In knee implants, the sensitivity and specificity of MRI in diagnosing PJI range from 65% to 92% and 85% to 99%, respectively [14]. Similarly, in the hip, the presence of periosteal reaction, capsule edema, and intramuscular edema demonstrated a sensitivity ranging from 78% to 95% and a specificity from 86% to 97%, depending on the signs that are considered for the diagnosis [23,50]. MRI also has the great advantage of not using ionizing radiation or contrast agents [24,25,26]. MRI may be limited by patient claustrophobia or the presence of an implanted non-MR compatible device.

### 5.5. What Is the Role of Three-Phase Bone Scan to Diagnose a PJI (Is a Negative Scan Sufficient to Exclude a PJI)?

Three-phases (perfusion, blood pool, osteometabolic phase) are necessary to perform a ^99m^Tc bone scan in suspected PJI. A positive bone scan may be observed in many conditions characterized by increased osteoblast activity, and therefore it is not specific for infection; a negative scan in all three phases means that there is no increased perfusion and no increased osteoblastic activity. Given its high negative predictive value (NPV), a negative three-phase bone scan is sufficient to rule out infection [14,53].

### 5.6. What Is the Minimum Time Window between the Date of Surgery and a Three-Phase Bone Scan to Diagnose a PJI?

Within the first year after hip arthroplasty, periprosthetic uptake patterns are variable depending on the type of surgery and device [54]. For cemented hip arthroplasties, the majority of asymptomatic patients have a normal bone scan, but up to 10% will have persistent periprosthetic uptake after one year from implantation. For porous-coated hip arthroplasties, persistent uptake beyond one year is even more frequent. Furthermore, few data are available about the longitudinal evolution of normal periprosthetic uptake patterns around hybrid, bipolar, and hydroxyapatite-coated devices. Periprosthetic activity around knee arthroplasties in asymptomatic patients is present in more than 50% of femoral components and nearly 90% of tibial components more than one year following implantation. Although periprosthetic activity usually becomes milder over time, there is considerable variation among patients and therefore serial scans should be performed [55]. Since it is not possible to clearly define a date, it has been suggested that positive bone scans should be interpreted with caution for a period of two years from surgery for hip and shoulder prosthesis and a period of five years for knee prosthesis. On the other hand, it can be postulated that a negative bone scan virtually excludes a PJI even within the above reported time windows [53].

### 5.7. What Is the Role of a WBC Scan to Diagnose a PJI (Is a Negative Scan Sufficient to Exclude a PJI)?

Several systematic reviews and meta-analyses have been published indicating that WBC scans—if necessary, combined with a bone marrow scan—have very high specificity for identifying peri-prosthetic joint infection versus aseptic loosening [55,56,57,58,59,60], thus representing the most reliable imaging tool able to achieve this differentiation. The reported accuracy of the use of combined WBC scintigraphy (using either ^99m^Tc-HMPAO-WBC or ^111^In-oxine-WBC) and bone marrow scintigraphy ranges from 83% to 98% for both hip and knee prosthesis infections [34].

Expert opinions and most research studies indicate a high NPV for WBC scintigraphy. This could be even higher if the correct acquisition protocols and interpretation criteria are applied [30]. In fact, NPVs ranging from 92% to 100% have been reported in the largest and most recent studies. [31,56]. Therefore, we can conclude that a negative WBC is sufficient to exclude a PJI.

### 5.8. What Is the Role of [^18^F]-FDG-PET/CT to Diagnose a PJI (Is a Negative Scan Sufficient to Exclude a PJI)?

While there is considerable debate about the specificity of [^18^F]-FDG, most investigators agree that the test is very sensitive and therefore the negative predictive value is high [54,61,62]. In an investigation of 21 patients with suspected PJI of the knee, [^18^F]-FDG-PET was 100% sensitive, but only 73% specific for infection [63]. In a recent investigation in 130 patients with suspected PJI of the hip, with final diagnosis based on the criteria recommended by the MSIS, [^18^F]-FDG-PET/CT yielded a sensitivity and specificity of 95% and 39%, respectively, for infection [64]. Based on the available literature, it seems reasonable to conclude that a negative result effectively excludes PJI. Whether or not [^18^F]-FDG is superior to bone scintigraphy for excluding infection is not answerable with currently available data.

### 5.9. What Is the Spatial Resolution of Currently Available Imaging Techniques in Order to Describe the Extent of a PJI?

The spatial resolution of a planar WBC scan is approximately 0.8–1 cm and by SPECT, the spatial resolution is 0.5–0.6 cm. The newest digital PET/CT scanners can reach resolutions as low as 2–3 mm.

Morphological examinations such as CT and MRI have much higher special resolution as compared to nuclear medicine modalities.

However, available imaging techniques only reflect the extent of the host’s response, i.e., the inflammatory process, or describe morphological changes due to the interaction between the pathogen and the host. This does not necessarily reflect the extent of the infection. Accurate delineation of the extent of the infection around an implant would require an infection-specific imaging technique, which is currently lacking.

### 5.10. Can Clinicians Rely on a Scan to Decide to Maintain a Component of an Implant If Infection Is Ruled Out by Imaging Investigations?

If imaging modalities (radiological and/or nuclear medicine) exclude the presence of infection, clinicians can decide to maintain one or all components of the prosthesis mainly because all imaging modalities have very high sensitivity. In these cases, a component should be removed based on the degree of loosening and patient compliance. On the other hand, if an infection is suspected by imaging, to the best of our knowledge, there are no published data suggesting that infection can be limited to an individual component of an arthroplasty and that this can be reliably assessed by an imaging modality.

### 5.11. Is There Any Evidence That Imaging Techniques May Have Different Accuracy or Thresholds to Diagnose High-Grade and Low-Grade Peri-Prosthetic Joint Infections?

There are no comparative studies investigating the accuracy of imaging techniques in patients with high-grade, acute peri-prosthetic infections versus low-grade, sub-acute or chronic clinical presentations.

Nuclear imaging has been shown to be effective at differentiating chronic low-grade infection in painful knee prostheses with a sensitivity and specificity of 71% and 95%, respectively, for combined WBC/bone marrow scintigraphy [53].

However, the sensitivity of nuclear imaging techniques can be significantly reduced in low-grade, chronic PJI of the shoulder. In fact, remarkably poor sensitivity of both [^18^F]-FDG and combined labelled leukocyte/marrow imaging to diagnose chronic, low-grade periprosthetic shoulder infection has been reported, with respective values of 14% and 18% [65,66]. Since there are no data on “high grade” shoulder arthroplasty infections, it is impossible to determine if these results are related to the chronic/low grade presentation of PJI or if it is just a specific feature of shoulder PJI.

### 5.12. Are There Any Studies Comparing Intra-Operative Histological Findings and/or Microbiological Examination with Imaging Investigations?

In most investigations, final diagnoses are based on histopathology/microbiology. Overall, these studies have been summarized in several systematic reviews and were considered for preparing “evidence based guidelines” by EANM [14,16,30].

### 5.13. Is It Necessary to Stop Antibiotic Treatment before Performing a Scan to Diagnose a PJI?

It is not necessary to discontinue antibiotic treatment for a CT or MRI scan, neither for a bone scintigraphy or FDG-PET/CT. In contrast, it is believed that antimicrobial treatment may reduce the diagnostic accuracy of WBC scintigraphy, probably because of decreased number and activity of bacteria, which reduces the release of chemotactic factors, hence the accumulation of WBC at the site of infection over time. This accumulation over time is the physio-pathological principle on which the interpretation of WBC images is based.

However, there are only two studies in PJI [67,68] and one study in fracture-related infections using radio-labelled WBC [69] that show no differences in diagnostic accuracy between patients under antibiotics vs. antibiotic discontinuation. To the best of our knowledge, there is currently no data available on the impact of antibiotic treatment on the diagnostic performance of FDG-PET/CT in PJI.

### 5.14. What Are the Most Promising Technologies Currently under Investigation to Diagnose PJI and Other Implant-Related Infections?

Given the challenges in diagnosing PJI, an infection-specific agent would be very valuable. To achieve this aim, several attempts have been made using a variety of approaches including radio-labelled antibiotics, vitamins, sugars and peptides [70,71].

### 5.15. Should Nuclear Medicine Imaging Techniques Be Included in the Definition of Peri-Prosthetic Joint Infection and, in Case of a Positive Answer, Which One Would You Recommend?

Nuclear medicine imaging techniques should be included in the modern definition of PJI. In fact, these diagnostic tools, if adequately performed, provide an overall accuracy that can be considered similar to that of other commonly accepted examinations, or even better.

Unfortunately, despite several systematic reviews, meta-analyses and single studies, some clearly indicate that WBC scans—combined or not with bone marrow scans—are the most reliable imaging tool for identifying peri-prosthetic joint infection. Others suggest the use of FDG-PET or radio-labelled anti-granulocyte antibodies, or even bone scans [34,53,56,61,65,67,72,73,74,75,76,77,78,79,80,81,82,83,84]. This needs to be clarified. There is a considerable variation in results when looking at individual studies due to different labelling methods, image acquisition protocols, image interpretation, patient selection, etc. Furthermore, most systematic reviews do not include all published studies, nor a set time interval for paper selection. As a result, in some “systematic reviews” we find as little as three, or even two or just one, paper(s) on nuclear medicine modalities. In other meta-analyses, there are often a mixture of very old papers with very recent ones using completely different methodologies. Unfortunately, no recent large multicenter prospective multimodal comparative studies with standardized image acquisition and interpretation parameters exist, and therefore we cannot provide a firm evidence-based conclusion with regard to the imaging modality of choice for PJI.

Despite this, some practical considerations can be made. Indeed, in clinical practice, the choice of imaging modality will highly depend on local availability, waiting lists, patient claustrophobia, metal devices, operator experience and cost (which is country dependent). FDG costs approximately 150 €, takes approximately 2 h to perform, the waiting list is approximately 1–2 weeks, costs for the National Health Service (NHS) are approximately 1200 €, the radiation dose for the patient is approximately 6–8 mSv and the overall diagnostic accuracy for PJI ranges from 65% to 90%. A WBC scan (or anti-granulocyte antibodies) costs approximately 150 €, takes approximately 2 h for labelling (the patient is busy from 8:00 am to 3:00 pm on the first day and from 8:00 am to 9:00 am the following day), the waiting list is approximately 1–3 weeks, the cost for the NHS is approximately 450–1000 €, the radiation dose for the patient is approximately 5 mSv and the overall diagnostic accuracy for PJI ranges from 70% to 95%.

It emerges that there are pros and cons for both modalities. If we require a very urgent screening test, particularly in patients with a low pre-test probability of infection, we can perform a bone scan or FDG (both able to effectively rule-out a PJI when negative, but could show residual inflammation for a long time after surgery) [53,54,85,86,87]. If we know upfront that there is a high suspicion of infection, or if we do not know if there is an infection or an aseptic loosening, it is preferable to perform a WBC scan [14,16,30,88,89].

## 6. Conclusions

Several definitions of PJI exist, but the use of imaging modalities is lacking in most of these scoring systems.

In this manuscript, we focused on the current role of several different imaging techniques in order to understand if this exclusion is justified in light of their possible contribution to diagnose a peri-prosthetic infection.

The panel highlighted how several imaging techniques, their limits notwithstanding, may play a key role in PJI definition.

While *X*-ray examinations may currently be regarded as a general screening for patients with joint replacement, MRI and nuclear imaging techniques are much more specifically concerned with the differential diagnostic work-up of PJI.

Based on available data in the literature, three-phase bone scans, WBC scans and FDG-PET scans are all highly sensitive investigations; whenever negative, they can all be reliably considered as a criteria to exclude a PJI. Furthermore, a positive WBC scan (if necessary, combined with a bone marrow scan), is to be considered a confirmative criteria of PJI.

Concerning FDG-PET/CT, there is a need to establish clear and standardized interpretation criteria to differentiate infection from non-infectious pathologies, especially aseptic loosening.

Finally, although very promising and attractive for its preliminary results, easy accessibility and lack of ionizing radiation, MRI appears to be a potential important player; if further studies confirm its accuracy in diagnosing PJI, it may be another imaging modality that will need to be included in the upcoming PJI definitions.

## Figures and Tables

**Table 1:** Comparison of the diagnostic criteria adopted by five peri-prosthetic joint infection (PJI) definitions, published from 2011 to 2018 (modified from [13]). MSIS: Musculo-Skeletal Infection Society; IDSA: Infectious Disease Society of America; ICM: International Consensus Meeting; EBJIS: European Bone and Joint Infection Society.

Definition Source	MSIS 2011	IDSA 2013	ICM 2013	ICM 2018	Proposed EBJIS 2018
**Scoring System**	1 of the 2 major criteria OR ≥4 of 6 minor criteria ^1^	≥1 positive criteria ^2^	1 of the 2 major criteria OR ≥3 of 5 minor criteria ^3^	1 of the 2 major criteria OR minor criteria scoring ≥6 infected 3–5 possibly infected (“consider further molecular diagnostics such as next-generation sequencing”) <3 not infected ^4^	≥1 positive criteria
**Criteria**	Major: sinus tract communicating with the prosthesis; A pathogen is isolated by culture from at least two separate tissue or fluid samples obtained from the affected prosthetic joint Minor: (a) Elevated ESR (>30 mm/hr) and CRP (>10 mg/L) concentration (b) Elevated synovial leukocyte count (c) Elevated PMN% (d) Purulence in the affected joint (e) Isolation of a microorganism in one culture of periprosthetic tissue or fluid (f) Greater than five neutrophils per high-power field in five high-power fields observed from histologic analysis of periprosthetic tissue at 400× magnification	Sinus tract communicating with the prosthesis Purulence without other aetiology surrounding the prosthesis Acute inflammation seen on histopathological examination of the periprosthetic tissue ≥2 intraoperative cultures or combination of preoperative aspiration and intraoperative cultures yielding an indistinguishable organism (the growth of a virulent microorganism (e.g., *Staphylococcus aureus*) in a single specimen of a tissue biopsy or synovial fluid is also considered as indicative of a PJI)	Major: A sinus tract communicating with the joint Two positive periprosthetic cultures with phenotypically identical organisms, Minor: (a) Elevated ESR (>30 mm/hr) and CRP (>100 mg/L for acute infections; >10 mg/L for chronic infections) (b) Elevated synovial fluid WBC count (>10,000 cells/mL for acute infections; >3000 cells/mL for chronic infections) or ++ change on leukocyte esterase test strip (c) Elevated PMN% (>90% for acute infections; >80% for chronic infections) (d) Positive histological analysis of periprosthetic tissue (>5 neutrophils per high-power field in five high-power fields observed on periprosthetic tissue at 400× magnification) (e) A single positive culture	Major: Sinus tract with evidence of communication to the joint or visualization of the prosthesis Two positive growths of the same organism using standard culture methods Minor: (a) Elevated CRP (>100 mg/L for acute infections; >10 mg/L for chronic infections) or D-dimer (unknown threshold for acute infection; >860 ug/L for chronic infection) (score 2) (b) Elevated ESR (no role for acute infections; >30 mm/hr for chronic infections) (score 1) (c) Elevated synovial WBC count (>10,000 cells/mL for acute infections; >3000 cells/mL for chronic infections) OR leukocyte esterase (++ for acute and chronic infections) OR positive alpha-defensin (score 3) (d) Elevated synovial PMN% (>90% for acute infections; >70% for chronic infections) (score 2) (e) Single positive culture (score 2) (f) Positive histology (score 3) (g) Positive intraoperative purulence (score 3)	Purulence around the prosthesis or sinus tract Increased synovial fluid leukocyte count (>2000 cells/mL or >70% granulocytes) Positive histopathology Confirmatory microbial growth in synovial fluid, periprosthetic tissue, or sonication culture (“confirmatory microbial growth in periprosthetic tissue: if positive in ≥1 specimen in highly virulent organisms or ≥2 in low virulent pathogens; sonication culture considered positive if >50 colony-forming units/mL of sonication fluid.”)

Abbreviations: ESR: erythrocyte sedimentation rate; CRP: C-reactive protein; PMN: polymorphonuclear leukocytes; WBC: white blood cells; ++: positive. ^1^ PJI may be present if fewer than four of these criteria are met. ^2^ The presence of PJI is possible even if the above criteria are not met. ^3^ PJI may be present without meeting these criteria. ^4^ Proceed with caution in adverse local tissue reaction, crystal deposition disease, slow growing organisms.

**Table 2:** World Association against Infection in Orthopaedics and Trauma (WAIOT) proposed definition of peri-prosthetic joint infection (PJI). Pre- and intra-operative tests, classified according to their sensitivity and specificity and hence their ability to exclude (“rule OUT”) or to confirm (“rule IN”) a PJI. In parenthesis, the reference cut-off value considered here (modified from [13]).

	No Infection	Contamination	BIM	LG-PJI	HG-PJI
Clinical presentation	One or more condition(s), other than infection, can cause the symptoms or the reason for reoperation (e.g., wear debris, metallosis, recurrent dislocation or joint instability, fracture, malposition, neuropathic pain)		One or more of the following: otherwise “unexplained” pain, swelling, stiffness		Two or more of the following: pain, swelling, redness, warmth, functio laesa
(Number of positive rule IN tests)-(number of negative rule OUT tests)	<0	<0	<0	≥0	≥1
Post-operatively confirmed if	Negative cultural examination	One pre- or intra-operative positive culture, with negative histology	Positive cultural examination (preferably with antibiofilm techniques) and/or positive histology		
**Rule OUT Tests (Sensitivity > 90%) EACH NEGATIVE TEST Scores − 1 (Positive Rule OUT Test Score 0)**					
Serum		ESR (>30 mm/h) CRP (>10 mg/L)			
Synovial fluid		WBC (>1500/μL) LE (++) Alpha-defensin immunoassay (>5.2 mg/L)			
Imaging		^99m^Tc bone scan			
**Rule IN Tests (Specificity > 90%) EACH POSITIVE TEST Scores + 1 (Negative Rule IN Test Score 0)**					
Clinical examination		Purulence or draining sinus or exposed joint prosthesis			
Serum		IL-6 (>10 pg/mL) PC (>0.5 ng/mL) D-Dimer (>850 ng/mL)			
Synovial fluid		Cultural examination WBC (>3000/mL) LE (++) Alpha-defensin immunoassay (>5.2 mg/L) or lateral flow test			
Imaging		Radio-labelled leukocyte scintigraphy (if necessary, with combined bone marrow scintigraphy)			
Histology		Frozen section (5 neutrophils in at least 3 HPFs)			

Abbreviations: BIM: biofilm-related implant malfunction; LG-PJI: low-grade peri-prosthetic joint infection; HG-PJI: high-grade peri-prosthetic joint infection. ESR: erythrocyte sedimentation rate; CRP: C-reactive protein; IL-6: interleukin-6; WBC: white blood cell count; PC: procalcitonin; LE: leukocyte esterase strip (++); HPFs: high power fields (400×); ^99m^Tc: 99 metastable Technetium.
